# Policy Implementation Science to Advance Population Health: The Potential for Learning Health Policy Systems

**DOI:** 10.3389/fpubh.2021.681602

**Published:** 2021-06-17

**Authors:** April Oh, Ali Abazeed, David A. Chambers

**Affiliations:** Implementation Science Team, Division of Cancer Control and Population Sciences, National Cancer Institute, National Institutes of Health, Rockville, MD, United States

**Keywords:** implementation science, public health, health equity, population health, health policy

## Abstract

Many health policies are designed with the intention of improving health outcomes for all. Yet implementation of policies are variable across contexts, potentially limiting its impact on population health outcomes. The potential impact of a policy to advance health equity depends both on the design and its implementation, requiring ongoing evaluation and stakeholder engagement. Despite the importance of health policies in shaping public health, health care policy implementation science remains underrepresented in research. We argue that enhanced integration of policy questions within implementation science could reduce the time lag from policy to practice and improve population health outcomes to build a body of evidence on effective policy implementation. In this commentary, we argue that approaches to studying policy implementation science should reflect the dynamic and evolving policy context, analogous to the “learning healthcare system,” to better understand and respond to systematic and multilevel impacts of policy. Several example opportunities for a learning health policy system are posed in building a broader agenda toward research and practice in policy implementation science in public health.

## Introduction

Once health policies are developed and adopted, they do not implement themselves. Rather, they require monitoring, evaluation, and stakeholder engagement to achieve intended goals. As countries move into post-pandemic recovery, this commentary encourages researchers, policy stakeholders, and policy advocacy organizations to collaborate together to take a systems approach to policy implementation. The need for a systems approach in public health has been highlighted by the recent disruption of the COVID-19 pandemic, which illustrated the interconnectedness of social systems including the health system, employment, housing, public health, food system (1), and education systems, which intersect and can have ripple effects from one sector to another. For example, when schools and childcare facilities were closed, nursing and support care staff shortages in the health care system were reported due to lack of childcare. Indeed, it highlighted the urgency and the need for ongoing evaluation and monitoring of health related policies and programs, and their implementation in order for our public health and social systems.

Broadly, many health policies are designed with the intention of improving health outcomes. A recent cross-country analysis of state policies to advance health equity found great variability in policy implementation approaches by state (2). Investigators found that the potential impact of a policy to advance health equity depends both on the design and its implementation, requiring ongoing evaluation, and stakeholder engagement. This discourse suggests moving beyond sole focus on development of evidence-based policies to a systematic examination of how those policies are implemented. Each public sector service setting is governed by a range of policies enacted at national, state, and local levels, which affect access to quality care, health services workflow, and environmental factors affecting health within communities. The processes through which these policies are implemented will shape how successfully each setting can deliver effective services.

## Policy Implementation Science

Within health care, implementation of quality scientific evidence has an estimated lag from research to clinical practice of 17 years (3). Implementation Science is the study of methods that promote the uptake of research findings into routine practice. Despite the importance of health policies in shaping public health, policy implementation science remains underrepresented in research. We argue that enhanced integration of policy questions within implementation science could reduce the time lag from policy to practice and improve population health outcomes to build a body of evidence on effective policy implementation. A key example of an opportunity for policy implementation science is in the case of Medicaid coverage policies in 2010. Several states' Medicaid coverage policies expanded coverage to include cancer screenings. While these policies improved health access and observed higher Medicaid enrollment, higher cancer screening rates in low-income adults were not realized (4). One potential missed opportunity was to systematically assess variation in how policies were implemented across practices, as well as more explicitly utilize implementation science to effectively realize the population health goals of policies supporting cancer screening, behavior change, or improved clinical practice. In the current public health environment of the SARS-COVID-19 pandemic, as different states have implemented stay at home orders, health care delivery protocols, and vaccination communications, sharing how these policies are implemented and tracking their outcomes can help us better understand how effective strategies can enhance implementation and most importantly, reduce morbidity and mortality. In fact, because local policies continue to rapidly change and require evaluation, the NIH has invested in the RADx-UP initiative to identify and develop an evidence base for implementation of COVID-19 testing and testing in the context of vaccination (5).

Policy implementation science examines how governments and organizations move policies into effect (6), but knowledge gained may be limited without the use of iterative and cyclical models and methods to examine how policies are implemented. Consideration of this agenda requires tackling one of the biggest challenges in understanding healthcare policy context: policy's inherent dynamism and uncertainty. Changes to legislative bodies in each new election cycle brings new priorities, and the dynamic 24-h news cycle of the current US landscape shapes societal understanding and demand for services, which affects the policy agenda. This dynamic and uncertain policy context leads to changes in the healthcare environment, allocation of resources, and rapid adjustments to account for new laws and regulations.

This commentary outlines an opportunity for policy implementation science. We argue that approaches to studying policy implementation could embrace the dynamic and evolving context of our health care environment, analogous to the “learning healthcare system,” (7) to better understand and respond to the multilevel impacts of policy. This paper will highlight various examples from the US health care context to illustrate the potential for a learning health policy systems approach to support future policy implementation examination by policy researchers, implementation scientists, and key stakeholders and advocacy groups.

The development of a Learning Health Policy System (LHPS), enabling continuous policy implementation evaluation, could allow for an interdisciplinary and comprehensive examination of (1) how health systems respond and adapt to health policies, and (2) how health policies can shape health systems and the broader social and structural determinants of health. Current observational approaches that may view policy implementation as a discrete event are not responsive enough to capture the dynamics of our policy climate (e.g., elections, a rapidly evolving 24-h news cycle, and changing norms). Iterative approaches are suited to address this challenge of dynamism. A continuous systems approach, characteristic of a learning health system (8), would allow examination of these changes, and subsequent clinical and patient responses to policy, practices, and adoption. Feeding this information on implementation back into a learning system can enable policymakers to adjust and “retest” policy implementation and better respond to ongoing changes. We posit that a LHPS framework could be utilized as a way to engage a diverse array of organizations working with researchers, policymakers, and patient populations. The National Association of Community Health Centers (NACHC), The National Association of County Health Officials (NACCHO), and the Association of State and Territorial Health Officials (ASTHO) are just a few organizations that are tracking, evaluating, and advising on public health policy. Such organizations could collaborate with researchers to facilitate monitoring and evaluation of policy implementation including all stakeholders, establish common metrics, and identify evidence-based outcomes. Stakeholders may be motivated to adopt a LHPS to establish a monitoring and evaluation system that brings all stakeholders together in an iterative fashion during the implementation phase to assess whether or not goals are being met in real-time; whether or not policy processes need to change; and importantly, reduce the impact of negative or unintended externalities of a health policy. In the next section, we detail key components of the LHPS.

## Learning Health Policy System

The goal of a proposed LHPS is to dynamically generate evidence on policy implementation to inform adoption and sustainability of policy goals and outcomes for improved population health. We propose several components of a LHPS that leverage implementation science while also recognizing the evolving nature of policies themselves and their multilevel impacts (6).

**Figure 1:**
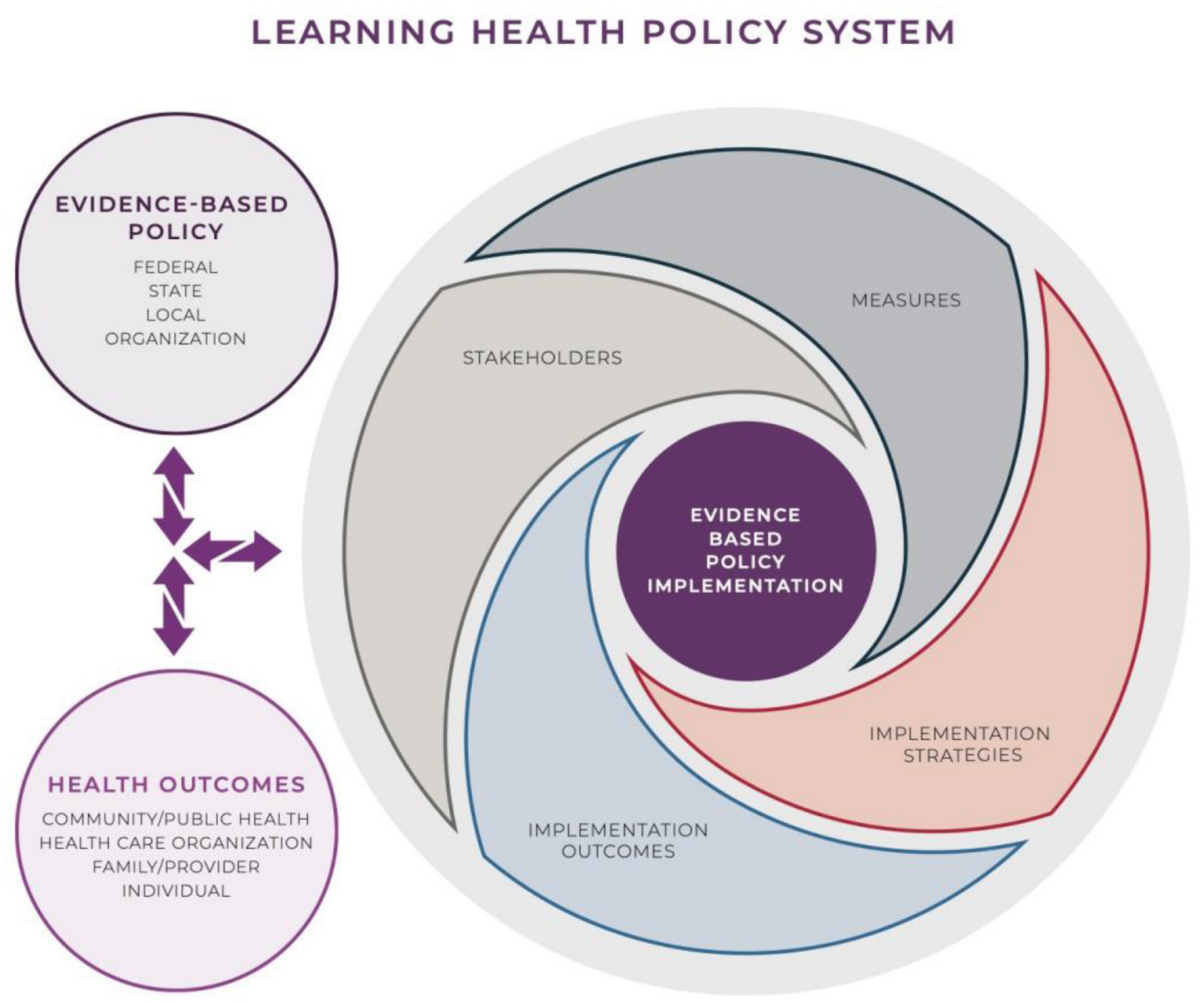
Learning health policy system.

Once a policy has been identified for examination for policy implementation in a LHPS (Figure 1) there are four important components. First, policy implementation should be studied in partnership with policy related stakeholders from top-down and bottom-up (patients, providers, health systems, cross sector partners, and policymakers). Recognizing effective health policy implementation requires the aggregation of separate actions of stakeholders, a LHPS would identify multi-level stakeholders to communicate objectives, ensure availability of resources, identify key policy implementers, manage conflict and cooperation, and sustain policy changes. Stakeholders are crucial in balancing biases and values, and having representation of multiple viewpoints will enhance the productivity of the LHPS, which will later have to be interpreted, communicated, and disseminated through the LHPS. For example, some studies have shown that a sample of policymakers working on health-related policies typically seek out evidence in the form of data and statistics (9), while other studies have categorized policymakers based on characteristics that may have implications for policy implementation (10). One such study showed that policymakers who are most influenced by budget impact are also most skeptical of behavioral health treatment effectiveness (11). Patients, on the other hand, are primarily concerned with access to primary care, self-care support, patient participation in clinical decisions, and partnering with their healthcare providers to make important decisions (12). As a field, Implementation Science has underscored the value of stakeholder engagement for accelerating implementation efforts and increasing the likelihood that data are useful, scalable, and sustainable in real-world settings (13). Partnership with stakeholders can help identify measures that allow the LHPS to capture ongoing policy effects and implementation strategies over time, working to reduce the time lag from policy enactment to implementation, resulting in health outcomes.

Second, measurement and data collection are essential to the formulation of a LHPS. Measurement should account for the ability to collect dynamic data, and to enhance external validity, mixed methods approaches may offer the ability to examine multilevel influences. Transdisciplinarity, pulling metrics from the intersection of public administration, organizational and individual behavior, implementation science, and political science may facilitate comparable multisector metrics that track determinants of change and policy characteristics that will affect the process and impact of policy implementation. Measurement can be ground truthed through qualitative examination, especially as multisectoral influences of policy on structural social determinants of health can be tracked. The iterative and systems approach in a LHPS require that metrics include both cross-sectional as well as longitudinal measures.

The data captured from these metrics can determine if policies are achieving their desired effect on health, specifically tracking the impact of the third element of an LHPS—implementation strategies. Implementation strategies are the techniques used to enhance the uptake and sustainability of the policies. These are an integral part of studying the policy itself. Some potential questions include:

- Are implementation strategies accounting for barriers to policy implementation?
- How do implementation strategies operate to facilitate adoption and sustainability of a policies intended effect?

Finally, the cycle continues as policy implementation outcomes can then be shared with stakeholders, specifically recognizing the influence on health outcomes. A LHPS is designed to be iterative, collecting implementation data to support rapid-cycle testing of policy implementation for translation of outcomes that are consistent with the policy's intended goals. A learning health policy system that includes an emphasis on outcomes that are communicated back into the system for evaluation and improvement is a departure from traditional policy evaluation approaches. Each element informs and influences the others, and evaluation and transparency across the processes facilitates dissemination of the knowledge learned for use in subsequent policymaking.

## Benefits of Using a Learning Health Policy System

The LHPS can facilitate examination of the intersection between health policy and implementation science, where policy occupies three specific roles:

- Health policies influence the context in which health interventions are implemented;
- Health policies are innovations implemented to improve population health; and
- Health policies provide a strategy to implement health interventions.

The following illustrates a few examples within the US health policy context of opportunities for a LHPS approach to policy implementation science.

A LHPS could advance understanding of how the health policy context shapes health interventions. Consider the Vaccines for Children program, a programmatic policy providing vaccine coverage for eligible children in the US. This policy reduces cost barriers to vaccination, impacting health systems' implementation of other evidence-based strategies to increase vaccination, such as provider recommendation interventions to promote vaccination (14). As policies impact health services contexts, data from LHPS metrics would quickly evaluate and disseminate best practices and provide information on how policies are influencing other practices. This type of system could be essential public health infrastructure, particularly during, and in post-pandemic planning and recovery. For example, while evidence continues to grow, disparities in COVID-19 incidence and mortality have been well-documented by racial/ethnic groups (15) with some studies indicating that enhanced availability and accessibility to SARS-CoV-2 testing and treatment could be more targeted in medically deprived areas (16). Multiple policies such as the Families First Coronavirus Response Act (17) or the Federal Coronavirus relief programs (CARES Act) have provided support and research funding to reduce barriers in receipt of COVID-19 testing and related services. A LHPS could identify and establish an evidence base for strategies that most effectively reduce or eliminate these barriers as well as a system for evaluation of policy implementation of vaccinations across the various modalities of vaccine rollouts to the public (18).

A LHPS also examines health policies as interventions, examining their direct impact on population health. Implementation Science models typically approach policy as an external influence on the provision and receipt of healthcare and a control variable in analytical models. In reality, policy can directly affect patients, providers, and health system outcomes. A 2018 trial of Healthy Behavior Incentive Programs (HBIPs) in Medicaid tested the use of financial incentives to encourage positive behavior changes (e.g., smoking cessation, weight loss) and reported little, if any, positive association with key health behaviors in the first 2 years of implementation. Post-implementation evaluation identified implementation challenges: poor awareness and adoption of the program, failure to properly communicate complex policy changes, and program delivery adaptations that reduced effectiveness (19). As identified in Figure 1, engagement of policymakers in the implementation process and building infrastructure responsive to policy could improve policy implementation decisions (20). This example underscores the importance of an iterative consideration of implementation strategies to achieve the desired effect of a policy change that helps bridge the policy to practice gap.

A LHPS could capture evidence to bridge the gap between policy and implementation science by examining how health policies drive implementation of evidence-based health interventions. The 2009 Health Information Technology for Economic and Clinical Health (HITECH) Act provided a $40 billion investment to promote adoption and meaningful use of Electronic Health Records (EHRs). More than 90% of hospitals and physician practices have adopted EHRs, and there has been growth of clinical decision support tools within EHRs to promote evidence-based clinical practice. In this case, a LHPS might identify key implementation strategies to drive deliberate change in health care practice, and we would have benefitted from iterative assessment of how EHRs affected intervention implementation (21).

Policies that change reimbursement for healthcare services, healthcare workforce requirements, healthcare credentialing and licensing, and regulation of sales may function as implementation strategies for health and healthcare improvement. For example, the COVID-19 pandemic has necessitated a shift in healthcare delivery resulting in stakeholders rapidly deploying and increasing their reliance on telehealth, an alternative to in-person care. Telehealth can facilitate access to care, reduce risk of transmission, conserve scarce medical supplies, and reduce strain on health care capacity and facilities while supporting continuity of care (22). During the COVID-19 Emergency, CMS issued temporary measures to make it easier for people enrolled in Medicare, Medicaid, and CHIP to receive medical care through telehealth services during the duration of the Emergency. CMS eliminated geographic restrictions and enhanced reimbursement so that telehealth services–enabled health centers could expand telehealth services and continue providing care during the pandemic (23). This shift in reimbursement policy during a public health emergency is a case study of a reconfiguration of time-limited policies. As shown in Figure 1, identifying and establishing common metrics among stakeholders is a key feature of the implementation process. Using a LHPS framework would allow examination of how policy implementation varies state to state; and collect measures and data that can be inform the LHPS. This process would allow examination of the effect of different implementation mechanisms on outcomes such as access, receipt of care, and quality of care. This systems approach would allow for rapid assessment of how such policy changes impact provider and patient behaviors as well as the implementation strategies that maximized patient benefit.

In contrast to prior efforts to promote evidence-based policy, a LHPS may offer policy implementation science a systematic, methodical approach to better understand and facilitate processes for realizing the population health goals of health policies. Health policy research frameworks have argued against the limiting assumptions of a “one size fits all” approach and called for integrating health across sectors to be reflective of the social determinants of health while promoting cross-sector partnerships (24). A LHPS may offer opportunities for a collaborative approach to improving the health of all people by incorporating health considerations into decision-making across sectors and policy areas. A learning health policy system can be challenging to operationalize, but the concepts presented here could be considered as methods toward shaping policy implementation science. It's important to note that while much of this commentary focuses on policy implementation, a LHPS also offers the opportunity to identify when policies may need to be deimplemented. For example, hypotheses and stakeholder concerns on whether disparities or inequalities are inadvertently being exacerbated by implementation processes that could do more harm than good.

## Conclusion

To advance studies of policy implementation, partnerships between implementation scientists, health service delivery systems, and policymakers around the LHPS concept offer great potential to generate important research and ensure a timelier pathway from research to practice. Past successful policy implementation shows the benefit for learning across a policy system. As observed in tobacco policies over the past 25 years, policies can evolve as they spread over time. For example, smoke-free policies started with the intention of preventing fires and food contamination and have now resulted in laws in half of US states requiring workplaces, restaurants, and bars to be 100% smoke-free. Such variation in implementation across states illustrates the potential for the LHPS approach where stakeholder input and common metrics could inform development and evaluation of strategies for other smoke-free practices to facilitate more rapid policy implementation. Effective policy implementation of smoke-free policies has reduced heart disease morbidity and lowered rates of hospital admissions for cerebrovascular accidents and respiratory disease (25). This example of effective policy implementation can serve as a model for studying how policy implementation can improve population health for other disease-related outcomes.

The window of opportunity to build a policy implementation science infrastructure is now, as policy changes and implementation are happening at an unprecedented rate in response to the SARS-COVID-19 pandemic and recovery planning. A systematic approach such as the LHPS offers the potential to include stakeholder engagement, account for the iterative and dynamic nature of policies in response to the pandemic and respond to growing social and structural inequalities.

## Data Availability Statement

The original contributions presented in the study are included in the article/supplementary material, further inquiries can be directed to the corresponding author.

## Author Contributions

All authors contributed to manuscript revision, read, and approved the submitted version.

## Conflict of Interest

The authors declare that the research was conducted in the absence of any commercial or financial relationships that could be construed as a potential conflict of interest.
